# Synorth: exploring the evolution of synteny and long-range regulatory interactions in vertebrate genomes

**DOI:** 10.1186/gb-2009-10-8-r86

**Published:** 2009-08-21

**Authors:** Xianjun Dong, David Fredman, Boris Lenhard

**Affiliations:** 1 Computational Biology Unit, Bergen Center for Computational Science, University of Bergen, Thormøhlensgate 55, N-5008 Bergen, Norway; 2 Sars Centre for Marine Molecular Biology, University of Bergen, Thormøhlensgate 55, N-5008 Bergen, Norway; 3 Current address: Department for Molecular Evolution and Development, Centre for Organismal Systems Biology, Faculty of Life Sciences, University of Vienna, Althanstrasse, 1090 Wien, Austria

## Abstract

Genomic regulatory blocks are chromosomal regions spanned by long clusters of highly conserved noncoding elements devoted to long-range regulation of developmental genes, often immobilizing other, unrelated genes into long-lasting syntenic arrangements. Synorth  is a web resource for exploring and categorizing the syntenic relationships in genomic regulatory blocks across multiple genomes, tracing their evolutionary fate after teleost whole genome duplication at the level of genomic regulatory block loci, individual genes, and their phylogenetic context.

## Abstract 2

Synorth is a web resource for exploring and categorizing the syntenic relationships in gene regulatory blocks across multiple genomes.

## Rationale

A genomic regulatory block (GRB) is a chromosomal region spanned by an array of highly conserved noncoding elements (HCNEs; for other names of these elements see [1]). The span of HCNEs defines the extent of the block: in mammalian genomes the mean size of GRBs is estimated to be 1.4 Mb (median 1 Mb) [2]. HCNEs typically cluster around one particular gene in the region, most often encoding a transcription factor involved in the regulation of embryonic development and differentiation, referred to as the GRB target gene. Many HCNEs have been shown to act as long-range enhancers of the target gene [3-7], regardless of whether they are found within the target gene, close to it, or hundreds of kilobases away in either direction. In most cases, the target gene itself spans only a small fraction of the total GRB size. Often, much of the rest of the GRB consists of HCNE-spanned gene-free regions called gene deserts [8]. However, many GRBs also contain one or more unrelated genes, referred to as the GRB bystander genes, which often contain HCNEs in their introns and beyond but do not seem to be regulated by them. Instead, many of those HCNEs were shown to regulate the GRB target gene [9]. As enhancers, HCNEs must be in *cis *to (that is, within the response distance of) their target gene. As long as the function of the target gene depends on the regulatory inputs from HCNEs located within or near bystander genes, those genes are also locked into *cis *arrangement with the target gene. Indeed, we have shown that GRBs form the most ancient and most resilient regions of conserved gene order (synteny) across vertebrates [9], and across dipteran insects [10], as a result of the selective pressure that keeps the HCNEs in *cis *with the target gene. The conservation of synteny with near-perfect colinearity of HCNEs at the locus is an important defining feature of GRBs.

The key evolutionary mechanism that has the ability to affect the synteny and integrity of a GRB and its gene content is whole genome duplication (WGD). Immediately after WGD, the affected organism is a tetraploid - all its genes (and GRBs) are present in two copies per gamete. This duplicated genome content is highly redundant, so a WGD is followed by an extended evolutionary period during which one copy of most genes will become inactivated and disappear by neutral mutation - a process known as re-diploidization. A smaller fraction of the genes will remain in two copies that over time will either each specialize to perform complementary subsets of functions of the ancestral gene (subfunctionalization), or one will acquire a completely new function (neofunctionalization) [11].

Since each GRB (with the full set of target genes, bystander genes and HCNEs) is present in two copies following WGD, we say each has a 1-to-2 orthologous relationship with the ancestral (pre-WGD) genome. Over time, the aforementioned processes lead to inactivation of one copy of some of the GRBs (re-diploidization), reverting the orthology relationship with the ancestral genome to the 1-to-1 type. How we define the fate of a GRB is tied to the fate of its target gene(s): if the target gene survives in two copies, we consider that the GRB has survived in two copies ('1-to-2 scenario'); if, on the other hand, one copy of the target gene becomes inactivated, the HCNEs on that locus lose the gene on which they act and, as such, become non-functional, are no longer under selection, and are subsequently lost. This leaves the other GRB as the only copy in the genome ('1-to-1 scenario').

The bystander genes could also remain in two copies (1-to-2 orthology) or re-diploidize to a single copy (1-to-1 orthology). However, it is important to note that the fate and the final number of copies of each bystander gene can be, and often is, different from that of the target gene, and that the fates of different bystander genes in a single GRB are also different from each other.

Given the apparent independence of re-diploidization and/or subfunctionalization processes for each of the genes in a GRB, studying the number of copies of each gene, their location in the genome and the location of HCNEs can reveal many details about the evolutionary history of each GRB. For example, in 1-to-2 scenarios, the distribution of HCNEs between the two loci can help in the characterization of regulatory subfunctionalization of the two copies of the target gene [12]. As a special form of subfunctionalization, in the duplicated state there is a 'window of opportunity' in which it is allowed for one part of the HCNE array to break off from one copy of the GRB, as long as the equivalent part of the array is still in *cis *to the other copy of the target gene (for an example and detailed explanation, see Figure 7 in Kikuta *et al*. [9]). Additionally, the syntenic relationship between HCNEs and genes, and their locations after WGD, can reveal different mechanisms by which bystander genes escape synteny lock-in with the target genes. In ambiguous cases, this approach can help determine the actual target gene and infer boundaries between adjacent GRBs.

**Figure 1:**
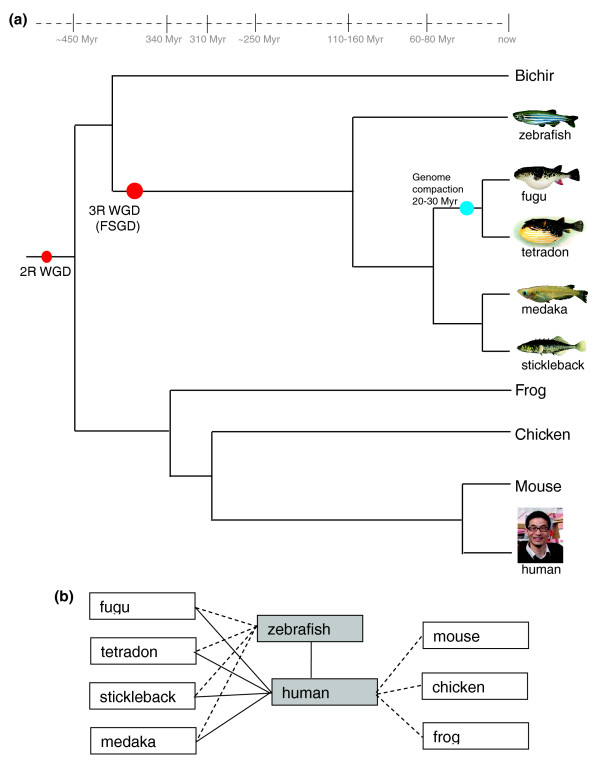
Species available for comparison in Synorth and their associated species tree. **(a) **Phylogenetic tree based on data from [14,50,70-72]. The red dots indicate the second-round (2R) and third-round (3R) WGD events [72]. The blue dot indicates the genome compaction in the pufferfish lineage beginning 20 to 30 Myr ago [70]. The species shown in the tree are: bichir (*Polypterus senegalus*), zebrafish (*Danio rerio*), fugu (*Takifugu rubripes*), Tetraodon (*Tetraodon nigroviridis)*, stickleback (*Gasterosteus aculeatus*), medaka (*Oryzias latipes*), frog (*Xenopus tropicalis*), chicken (*Gallus gallus*), mouse (*Mus musculus*), and human (*Homo sapiens*). Sources of fish images: Byrappa Venkatesh (fugu), Manfred Schartl (medaka), Wikipedia (zebrafish, tetraodon), Kraft CE *et al*. [73] (stickleback). **(b) **Reference and comparison species available in Synorth. Shaded boxes correspond to the reference genomes in Synorth. Connecting lines indicate genome pairs between which GRBs are available to check in the browser. Dashed lines indicate the genome comparison to be offered in the near future. The following genome assemblies underlie the current data sets: human NCBI 36, zebrafish Zv7 (The Wellcome Trust Sanger Institute), fugu v4.0 [74], tetraodon V7 [75], stickleback v1.0 (The Broad Institute), medaka v1.0 [51].

It is now established that there have been several WGDs in the course of evolution of chordates (Figure 1). The first round of WGD (the 1R WGD) is thought to have happened at the root of vertebrates around 550 Myr ago [13], after the separation from lancelets, hemichordates and urochordates. The 2R WGD took place at the root of jawed vertebrates. This is the last WGD in the human lineage, and many GRBs and their target genes were duplicated on that occasion (examples of duplicates from that event that remain in two subfunctionalized copies to this day are *SOX2/3*, *MEIS1/2*, *BARHL1/2*, *PAX4/6*). Extant jawless vertebrates (lampreys and hagfish) did not undergo this duplication, and their genomes will be used to compare the fates of GRBs after the 2R WGD once reasonably complete genome assemblies become available. The 3R WGD occurred 300 to 450 Myr ago, which is close to the root of today's teleost fish [14]. This is the WGD event that is the focus of the resource presented in this paper. Four teleost genomes have been assembled at the chromosome level (zebrafish, medaka, stickleback, tetraodon), and one at the level of large scaffolds (fugu). Since the genomes of other jawed vertebrates (including all tetrapods) did not undergo this duplication, they can be used with reasonable confidence as a reference for comparison that reflects the GRB structure of the last common ancestor before the 3R WGD. Indeed, there are almost no gross differences in general structure and gene content of the GRBs across tetrapod vertebrates, and any of their genomes may be used as a model for the ancestral structure of the GRB [15]. The 4R WGD might have happened as recently as 25 Myr ago in the ancestor of today's salmonid fish [16]. Since it was recent, the re-diploidization has not progressed far and the genomes of salmonid fish are still largely tetraploid [17].

There are two main tasks important for the interpretation of the impact of WGD and subsequent processes on the structure of GRBs: correct estimation of the extent of the GRB and distinguishing between the target gene(s) and the bystander genes in a GRB. With regard to the first, a GRB is defined physically by the extent of long-range regulatory elements around its target gene. Therefore, the combined synteny of HCNEs and the intertwined genes defines a minimal span of the GRB (see below for an approach we took to determine it genome-wide). The approach is not bulletproof as genes outside GRBs as well as multiple GRBs can still be syntenic by chance, and often are in more closely related species. In our experience, however, synteny estimation between human and zebrafish is a good conservative estimate of a GRB's span [15], even though a GRB may 'grow' by recruiting new regulatory elements at its edges after the separation of lineages. The new elements, however, do not help in elucidating GRB fate after WGD.

While there is no automated, failsafe method for distinguishing between the target gene(s) and the bystander genes in a GRB, there is a growing list of features of target genes that set them apart from bystander genes and other genes in the genome. These are: trans-dev function (most are transcription factors or co-factors, or developmental cell adhesion proteins); complex spatiotemporal expression pattern; long and/or multiple CpG islands; and distinct chromatin marks. For more details about each of these features of GRB target genes, see Akalin *et al*. [2] and Fredman *et al*. [18].

For an in-depth understanding of the concepts presented so far, the reader is advised to consult references [1,2,9,10,15,19], where detailed explanations and additional examples can be found. We have also prepared an animated introduction to the basic concepts, accessible from the Synorth home page.

With the emerging understanding of the GRB model, it has become clear that their study is inextricably bound to the WGD events in Metazoa, and that the most illuminating approach to studying their evolutionary history and the relationship between genes and their regulatory inputs should start with the analysis of syntenic relationships and re-diploidization scenarios following WGDs. A suitable tool for this type of analysis should enable the study of the evolutionary dynamics of HCNEs and gene content within GRBs, in the context of their genomic neighborhood and syntenic relationships. Here, we describe Synorth ("**Syn**tenic **orth**ologs") [20], a web-based application consisting of: a genome locus browser where all reference genome genes and HCNE locations in any given synteny block are displayed in relation to orthologous loci across multiple vertebrate genomes, with a number of adjustable parameters; a table browser that lists the orthologous and syntenic relationships for each bystander-target pair in a GRB, for each teleost fish species relative to human as a reference tetrapod genome; and a tree browser in which all genes in the GRB are projected onto an ideal gene tree that assumes a WGD event in teleost fish. We demonstrate how Synorth can be used to discover and visualize orthologous relationships, duplication and maintained synteny, and to trace genome rearrangement following the WGD. We anticipate that Synorth will also be useful for improving gene annotation and to visually detect genome assembly errors.

## A comprehensive ortholog dataset

**Figure 2:**
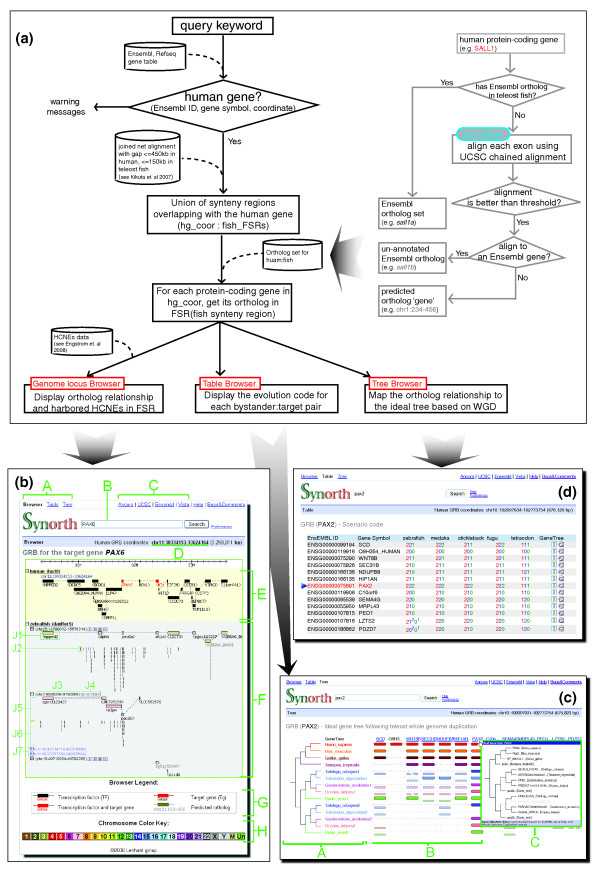
Synorth pipeline and three data views. **(a) **The Synorth data/analysis pipeline. **(b) **The Synorth genome locus browser: (A) navigation bar; (B) search field; (C) external link to other browsers (Ancora, UCSC Genome Browser, Ensembl and Vista) for the same region; (D) GRB genomic coordinates - the coordinates are the union of all synteny regions overlapping with the query gene - if no overlapping synteny region is found, no region will be shown; (E) reference species track; (F) track(s) of compared species - in each track, the species name and the sub-tracks for all synteny regions are shown; (G) browser legend; (H) chromosome color key for the compared species. For more details, see the description in the Help page of the Synorth website [20]. **(c) **Synorth table browser **(d) **Synorth tree browser.

To be able to study the evolution of HCNEs and gene arrangements in a genomic regulatory block context, we must first have a comprehensive and accurate annotation of gene orthology. We needed a comprehensive ortholog set that would be suitable for study of the evolutionary dynamics of genomic regulatory blocks, while considering a long-range regulatory model with gene loss, as well as difference in evolutionary rates among species [21]. This required an extension of existing methods for orthology detection to increase the coverage and assignment of mis- and un-annotated genes in incompletely annotated teleost genomes. To this end, we developed a strategy that combined Ensembl ortholog genes with ortholog genes predicted by an exon alignment pipeline (Figure 2a), and an examination of conserved synteny. Since we gave precedence to the Ensembl ortholog set, an ortholog predicted by exon alignment was used only if a gene did not have any orthologous genes in Ensembl (Additional data file 1).

In the final implementation, Synorth uses the Ensembl ortholog set, with two additional options that can be turned on or off: inclusion of additional orthologs predicted by our exon alignment pipeline; and exclusion of out-paralogs (paralogs whose origin predates the last common ancestor of the compared set of species; Additional data file 1). Inclusion of additional predicted orthologs improved coverage by providing orthologs for 424 out of 1,982 putative bystander genes in our initial GRB set that were missing in the Ensembl ortholog set (Table 1). By default, Synorth includes orthologs predicted by exon alignment and excludes out-paralogs (Additional data file 2).

**Table 1:** Ortholog gene counts in Synorth

	**Count of human orthologs detected in fish**				
	
**Source**	**Zebrafish**	**Fugu**	**Tetraodon**	**Stickleback**	**Medaka**
**Ensembl**					
One2one	7,790	8,429	7,784	8,903	8,639
One2many	6,144	6,285	7,718	5,868	5,307
Many2many	2,711	1,410	1,541	1,592	1,311
Apparent_one2one (out-paralogs)	197	192	181	175	238
Total	16,842	16,316	17,224	16,538	15,495
**Ensembl + Option 1 **(include exonAlign predictions)	26,695	20,529	20,460	23,070	21,269
**Ensembl + Option 2 **(exclude out-paralogs)	16,645	16,124	17,043	16,363	15,257
**Ensembl + Option 1 + Option 2 **(Synorth default set)	20,036	18,427	18,094	19,449	18,236

Counts for the different ortholog classes in the Ensembl ortholog set, the Synorth exon predictions, and out-paralogs that can be excluded via options on the Synorth preference page (Additional data file 2). Option 1 represents the inclusion of additional ortholog predictions from our exonAlign pipeline. Option 2 represents the exclusion of out-paralogs. The exonAlign ortholog prediction pipeline and the method used to exclude out-paralogs are described in Additional data file 1. The Ensembl ortholog set was extracted from Ensembl Compara version 49.

## Exploring genomic regulatory block evolution with Synorth

Users can explore GRB content and evolutionary rearrangement in three different modes (Genome locus browser, Table browser, and Tree browser) through the links in the top-left corner of the Synorth start page [20]. The Genome locus browser shows GRB genes and HCNEs in the reference genome in a locus-centered genome browser fashion, and additionally shows multiple tracks for each compared species (Figure 2b). The Table browser describes the evolutionary fate of each bystander gene in the GRB using the scenario code we developed for this purpose (Figure 2c). The Tree browser shows GRB rearrangement(s) among species in the context of an ideal gene tree in a simplified cartoon form (Figure 2d). Synorth currently supports analysis of GRBs in human and fish genomes (zebrafish, fugu, tetraodon, stickleback and medaka; Figure 1b). We aim to expand this list in the future to study other perspectives or instances of WGDs, after the suitable genome assemblies become available. The first on the list is the upcoming Zv8 zebrafish genome assembly as a reference genome, followed by the lamprey genome for studying the 2R WGD.

### Genome locus browser

For any supported input query (gene symbol or reference (human) genomic location), the browser shows the region containing all synteny blocks overlapping with the input query (Figure 2a) and their orthologous content in the compared genomes, one genome per row. Each orthologous gene and HCNE is horizontally aligned to its human ortholog for quick visual assessment of retention, rearrangement and loss. The sizes of genes in the compared (fish) genomes are not drawn to scale, but are reshaped to keep the same spacing and length as in the reference genome and so align vertically with them (Figure 2b). Clicking on the gene models brings up gene information in the UCSC Genome Browser [22]. By default, the orthologs are colored by the chromosome on which they reside in the other genome. If the GRB content maps to more than one chromosome in the compared genome, each chromosome will be shown on a separate track, and the tracks are ordered by the number of orthologous genes they contain. To visualize the tendency of HCNE arrays to correspond to large synteny blocks, we also included tracks showing HCNEs between the reference genome and the compared genome (using the HCNE data from Ancora [15], with window size 50 bp and similarity threshold 70% for mammals:teleosts), which are displayed below the genes in each track. The browser also provides links that bring up the same synteny region in Ensembl [23], UCSC [22], Ancora [15] and VISTA [24] genome browsers.

### Table browser

**Figure 3:**
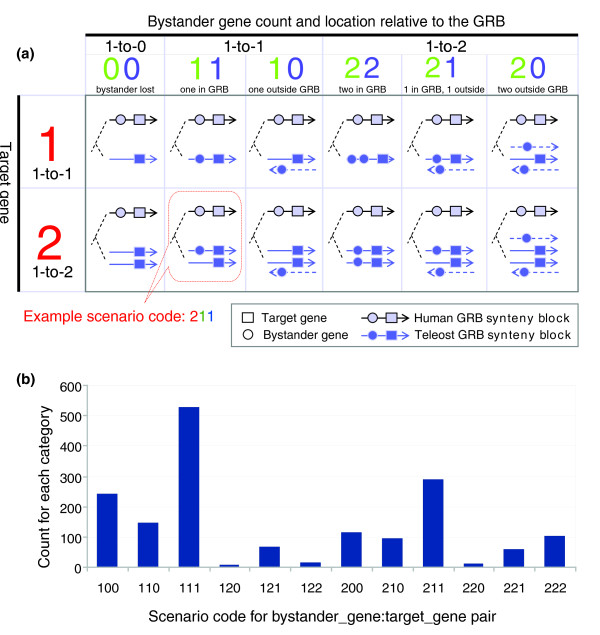
Scenario code illustration and statistics. **(a) **Definition of scenario code. **(b) **Descriptive statistics of scenario codes for all bystander-target pairs in 215 curated GRBs.

A GRB target gene, which is often a developmental transcription factor, is spanned by a synteny-maintaining array of HCNEs [25], many of which were shown to act as the gene's regulatory inputs [6,26], often intertwined with other, unrelated (bystander) genes [9]. To trace the fate of genes in GRBs after WGD relative to the reference genome (which we assumed to contain an ancestral arrangement of genes in GRBs [1] - see Rationale), we need to define the orthologous mapping positions of bystander genes in relation to the target gene. Here we define a code for each bystander-target gene pair, which is composed of three digits 'XYZ' (Figure 3a): the first digit of the code, X, represents the number of the target gene orthologs in the compared species (which also means it is a 1-to-X scenario for the GRB evolution); the second digit, Y, is the number of the bystander gene orthologs present in the compared species - Y can be 0 (not present in fish at all), 1 (re-diploidized bystander gene - one copy remains) or 2 (bystander gene survived in two copies); the third digit, Z, stands for the number of the bystander gene orthologs that are in synteny with the target gene (Z = Y). For example, code '221' indicates that it is a 1-to-2 scenario for the target gene (the target was retained in two copies), that the bystander gene has also been retained in two copies in the compared (fish) species, and that only one of the two copies of the bystander gene is still in synteny with the corresponding copy of the target gene in the fish genome.

The code captures the relationship of the bystander orthologs and in-paralogs [27,28] with the corresponding target genes, with respect to the copy number and synteny conservation. It is important to understand that the full three-digit codes refer to bystander genes and capture three important parameters of their fate with respect to the ancestral GRB they were part of. Since each target gene is, by definition, retained in the same number of copies as its GRB, and is still contained within all copies of the GRB, only the first digit has physical meaning for target genes.

The Synorth table browser shows the scenario codes for all bystander genes with respect to their target genes/GRBs in a table format, with one column for each compared species (Figure 2c). For each gene, a phylogenetic tree was built using TreeBeST [29] based on the multiple alignment of orthologs for human, mouse, chicken, frog and teleost fish. This tree building methodology is also used in Ensembl to build the protein family tree [23,30]. The trees can be accessed from links in the rightmost column of the table. For comparison, Synorth also provides the corresponding Ensembl protein family tree and ortholog tree from TreeFam [31,32].

### Tree browser

The tree browser is designed to reveal the evolutionary fate of genes at the level of the entire GRB (Figure 2d), instead of the single gene level as in the table browser. To construct this browser, we first projected the target gene tree (sub-tree from the Ensembl protein family tree) onto the ideal gene tree that includes the teleost WGD duplication. The synteny regions overlapping with the target gene's orthologs in each fish genome were recorded. Second, we mapped orthologs for each bystander gene within the GRB span onto their corresponding branches and levels in the ideal tree. In the ideal gene tree, each fish species has two branches, one for each of their duplicated gene paralogs. Each branch has two levels: the upper level (on the guide line) contains paralogs that are in synteny with the target genes; and the lower level (under the guide line) contains those paralogs not in synteny with the target genes (Figure 2d). For each bystander gene, any ortholog that was in synteny with a target ortholog was placed in the upper level of the same branch as the target ortholog. For each teleost bystander gene not in synteny with (either copy of) the target gene, we could map it to the correct branch when one of the other teleosts had two copies of the bystander gene, and both were in synteny with their corresponding target gene. In those cases, we compared the pairwise gene distances as measured by branch length in the gene tree, and defined the closer of the two genes in the other teleost as the ortholog. The bystander gene was then placed at the lower level of the same branch as that ortholog. The initial assumption for this method is that at least one ortholog in all the compared species is in synteny with the target gene; if neither was, they were placed in the tree in arbitrary order.

If the mouse pointer is hovered over an underlined gene name, a window showing the ideal gene tree for that gene pops up. Branches for which no ortholog genes were found in the tree are shown in gray, and are not underlined (Figure 2d). Paralogous branches of the same species are marked in the same color. The tree on the left side is the ideal gene tree for a perfect WGD model, which is based on the species tree in Figure 1a.

## Detecting the duplication, maintenance, and breakup of genomic regulatory blocks

As a result of WGD in teleosts, many mammalian GRBs have two orthologous regions in teleost genomes. Synorth makes it straightforward to visualize such mammal:teleost GRB orthologs by querying for a gene or genome region that overlaps with the GRB in the reference genome. For example, when viewing a human:zebrafish GRB, the synteny block (if any) spanning the human gene will be shown, and all its duplicated segments (if any) will be shown in zebrafish. One of the hallmarks of GRBs is that they are HCNE-dense regions [9,10], and that HCNEs aid in defining the extent of synteny across GRBs. We obtained HCNE track data from Ancora [15] and used them in Synorth both for analysis purposes and as a guide for visualization. By default, the browser displays tracks with more than ten HCNEs, tracks from chromosomes containing at least half of the GRB gene orthologs, tracks from chromosomes that harbor the ortholog of a transcription factor gene in the region, and/or tracks from chromosomes that harbor the predicted target gene. Users can choose to show/hide each track, or use one of the preset configurations available in the preference page (Additional data file 2). Figure 2b shows an example of the GRB for *PAX6*, a transcription factor gene with important functions in development of, for example, the eye, central nervous system and pancreas [33-35]. The GRB covers more than 2 Mb, harboring several bystander genes and an array of regulatory HCNEs [7]. Most of the human-zebrafish HCNEs in this region align to the orthologous loci of *PAX6 *on zebrafish chromosome 25 (*pax6a*) and chromosome 7 (*pax6b*). The bystander genes in the GRB are either present in a single copy on one of the branches (for example, *DPH4 *has one ortholog on zebrafish chromosome 25, and *elp4 *is left only on chromosome 7) or have disappeared from the zebrafish genome altogether (for example, *DCDC1*, which is highly expressed in human testis [36]). Thus, the browser quickly suggests that the noncoding putative regulatory sequences have been conserved to a similar extent at both of the duplicated *pax6 *loci in zebrafish, and that the bystander genes have largely re-diploidized.

**Figure 4:**
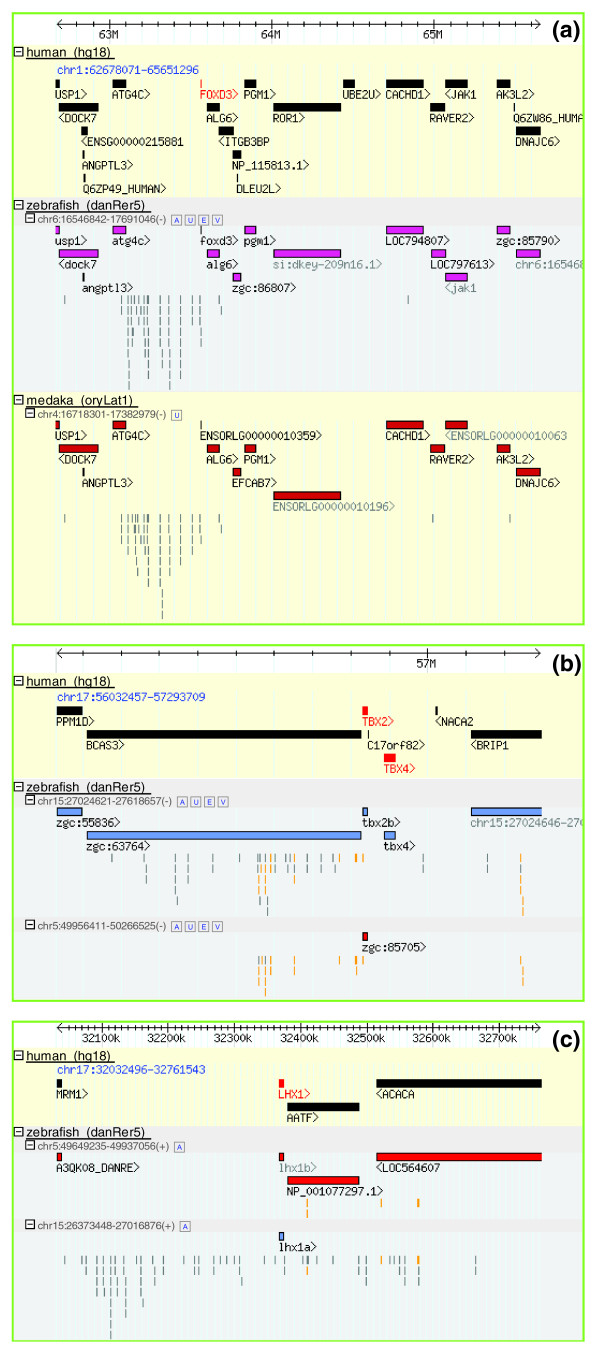
Using Synorth to show GRB duplication, maintenance and breakage. **(a) ***FOXD3 *locus, **(b) ***TBX2 *locus and **(c) ***LHX1 *locus. See the text for description.

In contrast, there are other cases in which target genes and the other GRB components (bystander genes and HCNEs) remain almost intact even after the WGD. Figure 4a shows an example for the GRB of *FOXD3*. Human *FOXD3*, a forkhead transcription factor gene upregulated in chronic myeloid leukemia, Jurkat T-cell leukemia and teratocarcinoma cell lines [37], lies within a GRB harboring a dozen other genes and a cluster of HCNEs, all mapping to a single syntenic locus in all teleost genomes (Figure 4a). A possible explanation for this is that one of the two copies of the entire locus was lost from the genome of a teleost ancestor by a large-scale chromosomal deletion shortly after WGD.

There are other, more complex cases that shed further light on the way GRBs and their components evolve. One of the more interesting scenarios is when a part of one copy of a duplicated GRB breaks off from its target gene. According to the GRB model, this is generally not tolerated in the ancestral (non-duplicated) GRB as it disconnects the target gene from a substantial number of its long-range regulatory inputs. However, after WGD, breaking off of a part of one GRB may be tolerated as long as the other copy of these disconnected regulatory inputs is still in *cis *to the other copy of the target gene [9]. For example, *TBX2*, a T-box gene encoding a transcription factor involved in the regulation of developmental processes in human [38-40] and zebrafish [41-43], is in the neighborhood of the gene *BCAS3*, and both are spanned by a cluster of HCNEs between human and zebrafish (Figure 4b). In zebrafish, *TBX2 *has two orthologous copies, *tbx2a *on chromosome 5 and *tbx2b *on chromosome 15. The ortholog of *BCAS3 *in zebrafish, *bcas3*, is still in synteny with *tbx2b *on chromosome 15, and contains a large array of intragenic HCNEs, with no other human:zebrafish HCNEs extending beyond that gene in that direction of the zebrafish GRB. In contrast, in the *tbx2a *zebrafish locus, only the 3' half of the corresponding HCNE array remains, and the zebrafish ortholog of *BCAS3 *itself is no longer present in that locus. The most parsimonious explanation for this arrangement is that there was a chromosome break somewhere in the middle of the zebrafish *BCAS3 *ortholog in the *tbx2a *locus, leading to the removal of the 5' part of the gene and that portion of the intragenic HCNE array. By this rearrangement, the remainder of the gene was non-functionalized and degraded through neutral evolution over time, while the intragenic HCNEs downstream of the break remained functional in *cis *to the *tbx2a *target gene, and were thus conserved.

Another scenario concerns GRBs with two copies of the target gene in teleost fish, where the two copies are surrounded by partially complementary sets of bystander genes and HCNEs. Figure 4c shows such an example, *LHX1*, a *LIM *homeobox transcription factor gene implicated in the development of head, nervous and reproductive systems [44]. It is apparent that the two zebrafish copies of the GRB harboring the *LHX1 *ortholog after WGD each lost alternative sets of HCNEs and bystander genes; the copy on chromosome 15 contains a large HCNE array and the target gene ortholog *lhx1a*, while the other branch on chromosome 5 contains all the other bystander genes, the target gene ortholog *lhx1b*, and very few HCNEs at the applied threshold. This differential pattern of HCNE retentionHCNE re broadly matches the complexity of the expression pattern of the target gene. While the expression of zebrafish *lhx1a *(synonym *lim1*; expressed in forebrain, hindbrain, neural tube and spinal cord [45]) corresponds well to the expression of the mouse gene *Lhx1 *[46], zebrafish *lhx1b *(synonym *lim6*) mRNA was found in lower amounts and in fewer spatiotemporal contexts compared to *lhx1a *mRNA [47], in line with an apparently lower number of regulatory inputs. The two zebrafish paralogs are expressed in complementary clusters of cells in the rostral telencephalon (Figure 7e, g in [48]).

From inspecting a number of cases such as the one above, it appears that the large-scale deletion of entire chromosomal segments (or possibly entire copies of duplicated chromosomes), as well as the event of one set of bystanders breaking off from their targets, could have been tolerated shortly after WGD while all genes and regulatory inputs on the other copy of the same segment were still fully functional. As time passed, more and more elements were selectively inactivated on either copy of the locus that still survived in two copies, making both essential for the full complement of their functions, and rendering further large-scale losses intolerable.

## Tracing the evolutionary change of genomic regulatory blocks among teleost fish

The WGD in the teleost fish lineage created two copies of most GRBs if the human genome is taken as outgroup of the teleost clade. Inspection of many of the loci reveals a striking confirmation and further explanation of the observation by Semon and Woolfe [49] that, in many cases, the fate of the GRBs after duplication is distinctly different in zebrafish and the other four fish - both with respect to the fate of individual bystander genes and whether the GRB and its target gene fall under a 1-to-1 or 1-to-2 scenario. This implies that the last common ancestor of zebrafish and the other four fish was still, to a large extent, tetraploid. Zebrafish is known to be an outgroup to the other four fish. For that reason, we suspect that some of the published estimates put the two events too far apart; for example, Wittbrodt *et al*. [50] state that the last common ancestor of medaka and zebrafish lived around 110 to 160 Myr ago (Figure 1a): since the teleost WGD is estimated to have occurred about 350 Myr ago, it would imply (rather implausibly) that much of the genome has remained tetraploid for more than 200 Myr, after which the reciprocal gene loss process took off. Other estimates [51] put the two events much closer to each other (WGD at 370 ± 34 Myr ago, zebrafish:medaka separation at 323 ± 9.1 Myr ago), which is more in line with what the interpretation of re-diploidization events would suggest. Synorth not only provides the most straightforward way to explore GRB content changes following the WGD, but also aims to visualize the differences in GRBs among the teleost fish, using human as a reference outgroup.

**Figure 5:**
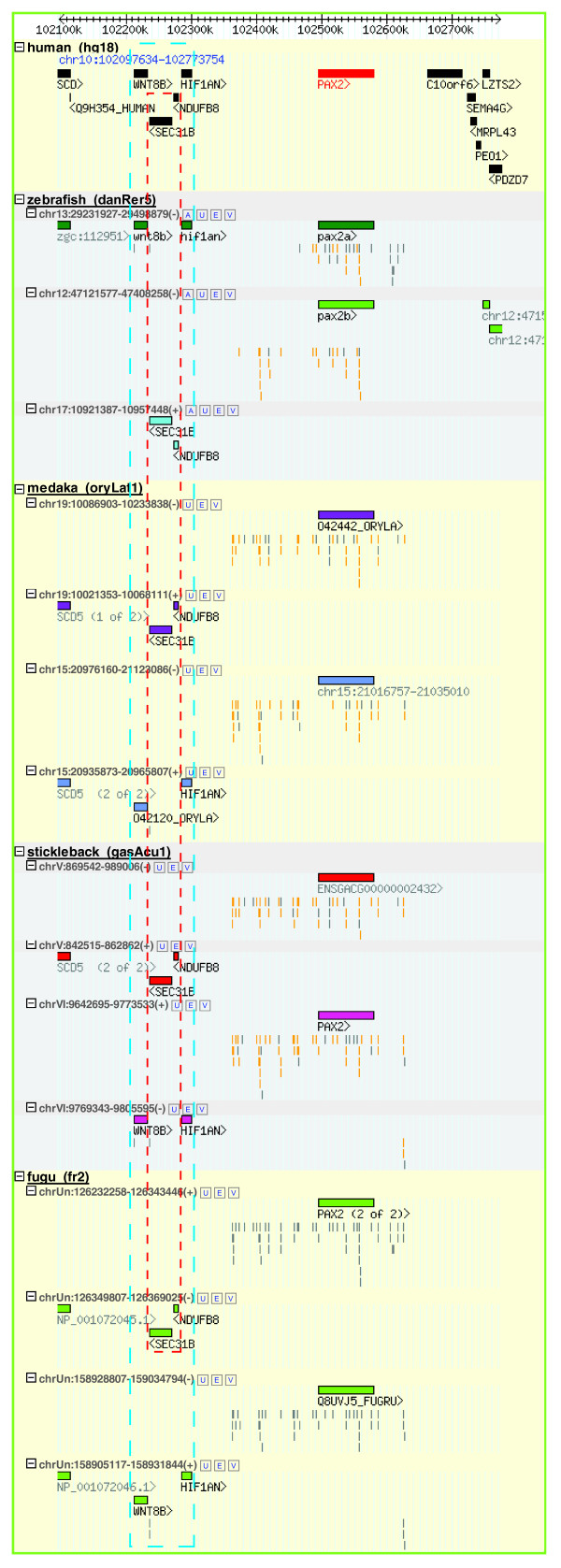
*PAX2 *example. Using Synorth to trace the GRB changes between teleost fish and to detect possible errors in genome assembly or missed annotation. See detailed interpretation in the text.

Figure 5 shows an example of how a GRB can change along with the speciation events on the fish species tree. The GRB for the candidate target gene paired box gene *PAX2 *contains a large cluster of HCNEs and several bystander genes. The *PAX2 *gene was found to play critical roles in eye, ear, central nervous system and urogenital tract development [52-54]. Several of the HCNEs that span the region around it were found to function as enhancer elements for its regulation [55]. The target gene has two orthologs in each of the five fish genomes (see below), each with an array of HCNEs that align to the single human *PAX2 *locus. According to the scenario code defined in the Table browser (Figure 2c), it is a 1-to-2 GRB. If we look at the four bystander genes upstream of *PAX2 *in order (*WNT8B*, *SEC31B*, *NDUFB8*, and *HIF1AN*), they are no longer syntenic as a group in teleost. *WNT8B *and *HIF1AN *are in synteny at one locus, and *SEC31B *and *NDUFB8 *in the other, showing a 'split-up' pattern (Figure 5). Interestingly, when we looked at their syntenic relationship with the target gene, zebrafish shows a different pattern than the other fish: while *wnt8 *and *hif1an *are in synteny with *pax2a *in all cases, from zebrafish to fugu, *sec21b *and *ndufb8 *are in synteny with the corresponding *pax2b *ortholog in all fish except the zebrafish. Again, this zebrafish outgroup feature is observed at many other loci, such as the GRBs for *FOXP2*, *SP3/SP5*, *MAB21L2 *and *TFAP2A*.

## Synorth as a tool for improving gene annotation, ortholog detection, and genome assemblies

Due to the fact that various duplication events, including WGD, have created multiple copies of many DNA segments in teleost genomes, gene annotation and genome assembly for teleosts has been shown to be difficult and error-prone. Using comparative genomics and phylogenetic methods, the approach taken by Synorth can aid in adding missing gene annotation and detecting likely cases of genome mis-assembly.

Returning to *PAX2 *GRB as an example (Figure 5), we can see that multiple HCNEs are present not only around zebrafish *pax2*, but around *PAX2 *orthologs in all teleost. In the medaka chromosome 15, the HCNEs and bystander genes are present, but without annotation of the orthologous *pax2*. This casts doubt on either the medaka gene annotation, or the target for HCNE regulation in this branch. From the medaka expressed sequence tag data in the UCSC Genome Browser [22] we could see that two unspliced expressed sequence tags map next to the orthologous position of *PAX2 *in medaka (chr15:21016757-21035010; data not shown). The situation is clearer in tetraodon; *PAX2 *seems to have an ortholog at chr2_random, supported by the GRB model and a high density of HCNEs there. The tetraodon assembly we used in Synorth (Ensembl 49, TETRAODON 7) has only one ortholog in chromosome 17: in the new assembly (TETRAODON 8), there is another ortholog, yet it is still in an unmapped contig (chrUn_random). Other cases of missed annotation, like the ortholog of human *PAX9 *in medaka, and the *ST18 *ortholog in zebrafish, could easily be corrected with the aid of the Synorth locus browser.

In addition to contributing to improved gene annotation, Synorth could also be used interactively to improve ortholog recognition. As shown previously, the ortholog prediction pipeline (the right hand-side of Figure 2a) used for Synorth outputs an extra set of orthologs ('prediction' in Table 1), which are shown in gray in the Locus browser. For example, in the *PAX2 *case, gene ENSGACG00000002432 (no gene symbol available) on stickleback chromosome V is in synteny with *SEC31B *and *NDUFB8*, spanned by an array of HCNEs, just like the case in most of the other teleost fish. The ENSGACG00000002432 gene was not predicted as an ortholog gene of *PAX2 *in Ensembl v49 (the version we used for prediction); however, Synorth provides ample evidence from synteny and HCNE content to annotate it as an ortholog. Cases like this were also mentioned in our previous examples, such as *lhx1b *- *jak1 *in zebrafish - which should be annotated as orthologs to their corresponding human gene according to Synorth and our ortholog dataset.

We have also found that Synorth could help in the detection and diagnostics of assembly errors in fish genomes by visualizing the problematic loci. An interesting case is that of *WWOX *(alleged bystander in the *MAF *GRB). The target gene, *MAF*, is a transcription factor that regulates differentiation, defects in which cause juvenile-onset pulverulent cataract [56]. There are two non-identical copies of *MAF *in zebrafish (Zv7), which are closely located on chromosome 18, while the single copy of *WWOX *is on another chromosome (chromosome 25), but still with HCNEs around it. According to the GRB model and the criteria for target gene selection (see Rationale and the cited references therein), these HCNEs should be associated with its target gene *MAF*, and not *WWOX*. This also appears true if we inspect the corresponding locus in other fish, where the synteny between the orthologs of *WWOX *and *MAF*, and the locus-spanning HCNEs, is intact. We checked this locus in the new Zv8 assembly by mapping the two *MAF *copies to it. Indeed, we found one of the copies mapped to chromosome 25, syntenic with the *WWOX *ortholog, as we expected, and the other mapped to chromosome 18 (Additional data file 3). This suggests that in the true zebrafish assembly, the first *MAF *ortholog (referred to here as *mafa*) should be placed on chromosome 25, in synteny with *wwox*, and the second one (*mafb*) on chromosome18, with both copies surrounded by HCNEs. This arrangement is similar to the orthologous loci in other fish. Other examples of possible genome assembly errors in zebrafish are in the loci of the orthologs of the human genes *GSX2*, *NKX2-4 *and *FEZF2*. They are presently 1-to-2 orthologs in human:zebrafish comparisons, but 1-to-1 in other fish genomes. In each case, the two zebrafish orthologs are closely located within one chromosome with very high sequence identity, but map to one locus in the new Zv8 assembly. This illustrates that, by considering the syntenic arrangement of corresponding loci among different genomes, Synorth can be used to detect a subset of likely assembly artifacts.

## Discovering prevalent evolutionary scenarios for genes in genomic regulatory blocks

As described above, we assigned a three-digit scenario code to each bystander gene that defines the rearrangement status of each bystander gene in relation to the target gene for the GRB in which it was located. This code offers a way to count the prevalence of different evolutionary fates for the contents of GRBs. Descriptive statistics for the scenario codes of all bystander:target gene pairs in the set of 215 curated GRBs that we investigated are shown in Figure 3b. Counting all bystander:target gene pairs for human:zebrafish belonging to the 1-to-1 scenario for the GRB, the top two scenario codes are '111' and '100' (Figure 3b). This means that most bystander genes in those GRBs were either maintained in synteny with the target gene, or not present in the zebrafish genome. In a few cases, the bystander gene was present outside of the orthologous GRB (group '110' in Figure 3b), where the zebrafish ortholog was present in a single copy, but no longer in synteny with the target gene. Those genes might have escaped the synteny with the target gene by reciprocal gene loss, leaving a copy of each in different loci [49]. For the genes belonging to the 1-to-2 orthology type, the most dominant scenario code was 211. This means most zebrafish orthologs of bystander genes were only present in single copy in the zebrafish genome, and that those orthologs were located in one of the orthologous GRBs. Scenario codes such as '210', '221', and '220', where the human bystander gene was found within the human GRB but outside of the zebrafish GRB, were found to be less common, most likely because such an arrangement requires a breakage/rearrangement of one copy of the zebrafish GRB, and this breakage can only occur in a 'window of opportunity' in which the corresponding part of the other copy of the GRB is still fully functional - that is, contains the full set of ancestral regulatory inputs.

## Summary

Synorth is designed to allow detailed study of the evolutionary changes in large chromosomal regulatory domains (GRBs) across vertebrate genomes. In its current form it is especially well suited for comparing changes in the different teleost fish lineages. Built upon a database of orthologous genes, syntenic regions and HCNEs, Synorth provides several ways of visualizing and summarizing the evolutionary changes of those syntenic orthologs in the context of HCNEs. One of its novel features is a straightforward way to display, measure, and explain the evolutionary changes of orthologous relationships in the framework of genomic synteny blocks. Ortholog relationships displayed in Synorth are qualitatively different from paired ortholog profiles available in other ortholog sets [23,32,57-61]: they clearly reveal regions of extensive noncoding conservation and highlight large chromosomal domains that have been maintained during evolution by the interaction of long-range regulatory elements and their target genes. Consequently, we anticipate that Synorth will be useful in tracing genes lost and gained in synteny regions, and for studying evolutionary events such as subfunctionalization following WGD. We have illustrated how Synorth can be used to visualize and explore the fate of orthologous genes through duplication, maintenance and breakage of GRBs. Synorth is also useful for improving ortholog recognition, gene annotation and genome assembly. The scenario code for bystander:target gene pairs defined in Synorth is also a powerful approach for the study of GRB evolutionary dynamics.

## Abbreviations

GRB: genomic regulatory block; HCNE: highly conserved non-coding element; Myr: million years; WGD: whole genome duplication.

## Authors' contributions

BL and XD designed the study. XD analyzed the data, created the Synorth web resource and the underlying database, and generated all examples and figures for the manuscript. DF co-supervised the design and development of the resource. XD, DF and BL wrote the manuscript.

## Additional data files

The following additional data are available with the online version of this paper: aupplementary methods (Additional data file 1); a figure showing the Synorth configuration page (Additional data file 2); a figure showing the use of Synorth to detect genome assembly errors (Additional data file 3).

## Supplementary Material

Additional data file 1 Supplementary methods. Click here for file

Additional data file 2 Users can set personal search preferences, including which species are used, the ortholog dataset, the appearance of the browser images (image width, connection lines, gene names, and so on), and set criteria that decide which tracks are shown by default based on track content ('smart' view mode). Click here for file

Additional data file 3 Using Synorth to detect genome assembly errors. Click here for file
