# Supplementary material for: Synorth: exploring the evolution of synteny and long-range regulatory interactions in vertebrate genomes
Source: Genome Biol. 2009 Aug 21;10(8):R86. doi: 10.1186/gb-2009-10-8-r86 (PMC2745767; doi:10.1186/gb-2009-10-8-r86)
Supplement: Additional data file 1 — Supplementary methods. [file gb-2009-10-8-r86-S1.doc]

# Supplementary note

## Synorth: exploring the evolution of synteny and long-range regulatory interactions in vertebrate genomes

Xianjun Dong ^1,2^, David Fredman ^1,2,†^, Boris Lenhard ^1,2,§^

^1^Computational Biology Unit, Bergen Center for Computational Science, University of Bergen, Thormøhlensgate, N-5008 Bergen, Norway

^2^Sars Centre for Marine Molecular Biology, University of Bergen, Thormøhlensgate, N-5008 Bergen, Norway

^§^Corresponding author:

Boris Lenhard

CBU, BCCS, HIB, Thormøhlensgate 55, N-5008 Bergen, Norway

E-mail: Boris.Lenhard@bccs.uib.no

Voice: +47 555 84362

Fax: +47 555 84295

^†^Current address: Department for Molecular Evolution and Development, Centre for Organismal Systems Biology, Faculty of Life Sciences, University of Vienna, Althanstrasse, 1090 Wien, Austria

Email addresses:

XD: xianjun.dong@bccs.uib.no

DF: david.fredman@univie.ac.at

BL: [boris.lenhard@bccs.uib.no](mailto:boris.lenhard@bccs.uib.no)

## Methods

### Definition of ortholog sets

There are several published orthologous gene sets, e.g. Ensembl orthologs [23], Evola [62], the OMA project [58], MSOAR [63], the KOG database [64], OrthoMCL [57], and InParanoid/MultiParanoid [61, 65]; however, neither of them alone is sufficiently complete and accurate to facilitate a comprehensive study of gene rearrangement following teleost fish (3R) WGD (Figure 1a). We required a set that defines orthology at the level of pairs (such as one-to-one orthologs, one-to-many and many-to-many orthologs, and paralogs). Ensembl orthologs, the collection closest to our requirements, uses the duplication score (S_dup_) of the common ancestor node for any given gene pair to define them as orthologs (S_dup_ = 0) or between-species_paralogs (S_dup_ > 0)[30]. However, we found that Ensembl’s stringent requirement of S_dup_ = 0 for orthology sometimes labeled well-supported orthologs as between-species_paralogs (e.g. human *PAX2* and zebrafish *pax2a/pax2b* in Ensembl v47 [66]). In only one other ortholog set, from the OMA project [58], orthology and paralogy were inferred at the level of pairs, but there many gene duplicates resulting from teleost (3R) WGD were not properly annotated (e.g. neither human *PAX2*:zebrafish *pax2a/b* nor human *PAX6*:zebrafish *pax6a/6b* were labeled as one-to-two orthologs in the OMA browser[58]). Most of the listed ortholog annotation methodologies are based on sequence similarity and/or phylogenomics. Where such methods give only weak support, one can exploit genomic linkage / synteny with genes whose orthology relationships are clearer, because genes close to each other in both species are likely to share duplication history. Other methods, like the SynBlast [59] and the commercial “syntenic-anchor” approach from Celera [67] can contribute to ortholog recognition by including the conserved synteny information around the focal reference gene.

We based our dataset of orthologous genes between various vertebrate genomes on the Ensembl ortholog set. To increase coverage, we then searched for orthologs for genes that lacked an Ensembl ortholog by an exon-alignment based strategy. We used pair-wise BLASTZ whole-genome chained alignments from UCSC Genome Browser database [68], and predicted an ortholog pair if the alignment of the gene reference genome sequence vs. the query genome covered i) ≥10 exons, ii) ≥50% of all exons, or iii) ≥50% of total transcribed nucleotides. The alignment status of an exon was defined as ‘aligned’ if ≥ 70% of the exon nucleotides were aligned in the chain alignment and ‘unaligned’ if < 15% nucleotides were aligned. Otherwise, the alignment percentage identity was returned.

An out-paralog is defined as a paralog that predates a species split (see Fig.1 in [28] for details), and is easily confused with true orthologs [27]. For Ensembl, we took the subset of orthologs labeled as “apparent_one2one” as an approximate set of out-paralogs [30, 69]. To resolve this issue for orthologs predicted by exon alignment, we built a phylogenetic tree using the TreeBeST method (http://treesoft.sourceforge.net/treebest.shtml) based on a multiple tBLASTn alignment for each human gene that had more than two orthologs in a teleost fish (e.g. zebrafish). By stepping through the tree from the node of the human reference gene up to the root node, we identified the minimal-length branch that contained the human gene node and its closest zebrafish ortholog, outside of which any additional apparent orthologs were counted as out-paralogs of the closest zebrafish ortholog.

We used the same method to extract the minimal sub-tree containing human, mouse, chicken, frog and the five teleost species from Ensembl protein family trees, on which the Tree Browser view in Synorth are based.

### Synteny blocks and GRBs

We identified human-teleost synteny blocks as described in Kikuta et al. 2007[9]. In brief, we based the synteny blocks on UCSC net alignments from teleost genomes to the human genome, and for each pairwise comparison counted the highest-scoring (level 1) net alignments as syntenic if 1) they were separated by ≤150 kb in the zebrafish genome and ≤450 kb in the human genome, and 2) they were on same strand. We kept the synteny block with the largest total amount of aligned sequence to the human genome in cases of block overlap in the zebrafish genome.

The region shown in the genomic locus browser in Synorth is the union of all synteny blocks between the reference species (i.e. human) and compared species (i.e. zebrafish, medaka etc.). If there were one (or more) candidate target gene(s) [2] in the synteny block (highlighted in red in the browser), we counted it as a GRB.

### References

The references are listed in the main text of the paper.
